# The Story behind the Science: Solving the riddle for the pathogenesis of vulvovaginal candidiasis

**DOI:** 10.1128/mbio.01787-26

**Published:** 2026-09-14

**Authors:** Paul L. Fidel, Junko Yano

**Affiliations:** 1 Department of Oral and Craniofacial Biology, School of Dentistry, LSU Health New Orleans 12258 , New Orleans, Louisiana, USA; Johns Hopkins Bloomberg School of Public Health, Baltimore, Maryland, USA

**Keywords:** *Candida albicans*, vulvovaginal candidiasis, inflammation, immunopathogenesis

## Abstract

For nearly three decades, the pathogenesis of vulvovaginal candidiasis (VVC) remained an enigma. During the HIV/AIDS epidemic, observations that women with profound CD4^+^ T-cell deficiencies were not more susceptible to VVC challenged the prevailing assumption that protective adaptive immunity dictated all mucosal *Candida* infections. Here, we recount the scientific journey and trajectory that began with these observations and culminated in a paradigm shift in understanding VVC pathogenesis. Through extensive clinical studies, animal models, and a groundbreaking live *Candida* challenge clinical study, VVC was ultimately redefined as an immunopathogenic condition characterized by a robust yet dysfunctional neutrophil response to *C. albicans* within the vaginal environment. We highlight the key discoveries, challenges, and experiences that reshaped the field and finally solved the long-standing riddle of VVC pathogenesis.

## COMMENTARY

This is the story behind how the Fidel lab solved the riddle underlying the pathogenesis of vulvovaginal candidiasis (VVC), a long-standing enigma that ultimately took almost three decades to solve. The story began in 1990 when I was appointed assistant professor in the Division of Infectious Diseases at Wayne State University in Detroit, MI, and began studying VVC along with Jack Sobel, chief of infectious diseases. The story continued at LSU Health–New Orleans beginning in 1995 in the Department of Microbiology, Immunology, and Parasitology, and subsequently at the LSU School of Dentistry for an additional 25 years. Over this 35-year period, the Fidel lab published 42 primary manuscripts summarizing data from the mouse model of experimental VVC (*n* = 34), a macaque model of experimental VVC (*n* = 1), and several clinical studies involving women with recurrent VVC (RVVC) (*n* = 7).

To initially study the host defenses against VVC, we established a mouse model that was an extension of a rat model that had been used primarily for drug testing (1–4), taking advantage of more immunological reagents available. Like the rat model, the mouse model is estrogen-dependent, with animals maintained in a pseudoestrus state before intravaginal inoculation with *C. albicans*, the primary causative agent of VVC (5, 6). Vaginal lavages are used to quantify fungal burden, and because immunological reagents were readily available for mice, the mouse model served as an excellent system to interrogate host defenses. Importantly, the mouse model also closely mirrored clinical VVC/RVVC by supporting persistent vaginal colonization with hypha-forming *C. albicans*, provided that the pseudoestrus state was maintained (7, 8). This remarkable similarity to human infection was observed despite several caveats of the animal model, including a neutral vaginal pH of rodents as opposed to the acidic pH of the human vagina (9, 10), the lack of a *Lactobacillus* species-dominated microbiome unlike humans (11), and the lack of endogenous *C. albicans* as normal commensal flora (12).

Identifying roles for adaptive immunity against VVC was critical in the 1990s as the HIV epidemic had revealed a dominant role for CD4 T cells in protection against oropharyngeal candidiasis (OPC) (13, 14), leading to the broad assumption that all forms of mucosal *Candida* infections were the result of some adaptive immune deficiency. At the same time, other groups working on vaginal candidiasis in the rat model and clinical cohorts reported putative protective roles for antibodies and T cells (15–17). This dogmatic view, however, was challenged by clinical observations that women with HIV infection, despite profound CD4 T-cell deficiencies and susceptibility to OPC, were not any more susceptible to VVC or RVVC (18, 19), which would have been expected if CD4 T cell immunity was the dominant protective mechanism against vaginal infection. Hence, a plethora of immunological studies needed to be performed, both in the mouse model and clinically, to confirm or deny these reports and, more importantly, to publish the findings to formally disseminate these data to the scientific community.

Over the next 10–12 years, through funding from NIH grants, we conducted several studies in the mouse model and in women with or without RVVC, characterizing the host immune response to *C. albicans* in the vagina. We evaluated the roles of systemic or local T cells, antibody-mediated immunity against infection, as well as any potential immune deficiencies associated with infection susceptibility. Because mice are not normally colonized with *C. albicans*, the host responses to infection could be easily monitored without any native immune interference from pre-existing *C. albicans*. Taking into account the existing dogma at the time, we found no role for T cells or antibodies in protection against VVC and no adaptive immune deficiencies in women with RVVC (20–25). Although these findings were largely negative, we were fortunate that reviewers and journal editors recognized their importance and supported the publications. However, we still had no evidence for what type of immunity was protective against VVC and how pathogenesis ensued. In retrospect, there was an important clue from the mouse model for the pathogenesis, but the significance was not realized until years later. The clue involved consistent observations of numerous leukocytes in the vaginal cavity during *Candida* vaginal infection. These cells were identified as polymorphonuclear neutrophils (PMNs), but since these PMNs failed to control or eliminate the fungal burden, we initially regarded them as simply part of the 4-day menstrual cycle for the mouse rather than a response to infection. However, in time, it became clear that the PMN infiltration was the hallmark of an immunopathogenic response that we ultimately interrogated as central to our understanding of VVC pathogenesis.

The critical study was conducted between 2002 and 2003 when we found ourselves at a dead end with the mouse model. We knew a great deal about what was not important in defense against VVC, but still understood very little about what was actually important in driving protective or pathological outcomes, with the exception of a modest, frontline protective role for epithelial cells that we showed could inhibit the growth of *Candida* through a contact-dependent fungistatic mechanism (26–30). Ultimately, after much deliberation, it was decided that a live challenge study in healthy women was needed to define the natural history of the infection and to identify any evidence of host responses/pathogenesis. We had previously attempted to characterize the host response in healthy women following intravaginal *Candida* antigen administration (25, 31). However, despite some evidence for a normal Th1-type response systemically, the results were not informative with little insight into the pathogenesis (25, 31). However, this study did provide a foundation from which to pitch the concept of a live challenge to the Institutional Review Board (IRB).

Upon submission of the protocol, the LSU IRB had many concerns. To address these concerns, we proposed a very conservative approach with a small inoculum administered to healthy women with no history of VVC. Women were also required to be free of vaginal *Candida* colonization and could self-treat with topical antifungals at any time if an infection ensued. We also emphasized that *C. albicans* is a normal commensal organism of human mucosal tissues, including the vagina, and that there was no evidence of any type of dissemination of *Candida* from the vaginal mucosa to the periphery (32, 33). Finally, we emphasized that while VVC negatively influences quality of life, no major illness is associated with infection (32, 34).

Ultimately, the IRB approved the protocol, and we embarked on the live challenge study. To our surprise, very few of the inoculated women contracted a symptomatic infection (30, 35). In most cases, any evidence of *Candida* in the vagina was cleared within a week. Hence, any concerns raised during the consenting process were short-lived as most women experienced no adverse effects. Likewise, long-term follow-up monitoring indicated no study-related adverse events. From there, we implemented a series of protocol amendments designed to improve the likelihood of symptomatic infection, including increased inoculum sizes, modifying the inoculum vehicle with glucose and/or exogenous estrogen, timing inoculation to the luteal stage of the menstrual cycle, inoculating with *C. albicans* hyphae rather than blastoconidia, and expanding enrollment to include asymptomatic women with detectable vaginal *Candida* colonization. Despite these modifications, the rates of symptomatic infection remained low. In total, only three of 19 women (15%) in this level 1 phase acquired a symptomatic infection. We, hence, amended the IRB further to broaden enrollment to women who had a previous history of VVC due to antibiotic use, oral contraceptives, or hormone replacement therapy (HRT) (level 2 phase). Alas, we finally began to observe symptomatic infection. More importantly, women who experienced symptomatic infection exhibited a massive vaginal leukocyte infiltrate composed almost exclusively of PMNs. Hence, the mouse model had indeed provided a clue for the pathogenesis via the vaginal presence of PMNs, providing the first direct clinical correlate of disease pathogenesis. As with most cases of VVC, symptoms were resolved following standard topical antifungal treatment. In the level 2 cohort (women with a prior history of VVC), symptomatic infection was documented in six of 11 (55%) women compared to only three of 19 (15%) women in the level 1 cohort (no history of VVC). But the most profound observation in all cases of symptomatic infection was the consistent presence of PMNs in the vagina, whereas women who remained asymptomatic showed little to no cellular infiltration. Although long-term follow-up identified no adverse effects, the biological role of these infiltrating PMNs remained a mystery. We originally planned to take the study one step further (level 3) and enroll women currently taking antibiotics or on oral contraceptives. However, ultimately we determined this was not necessary as the results from the level 2 cohort had provided sufficient evidence to propose new hypotheses regarding susceptibility and the pathogenesis of VVC/RVVC.

This seminal manuscript was published in 2004 (35) with the stated hypothesis that VVC pathogenesis was not associated with any immune deficiency, but rather an immunopathogenic response characterized by the recruitment of large numbers of PMNs into the vaginal mucosa. Yet, a perplexing question remained as to why these PMNs, despite their abundant presence, failed to reduce fungal burden or clear the infection. But nevertheless, this live challenge study opened the avenue for mechanistic studies in the mouse model to fully understand this newly identified immunopathogenesis.

Accordingly, over the next 15 years, we interrogated the mouse model to understand the mechanisms associated with the immunopathogenic response to VVC. These studies used disparate mouse strains, including several mouse strains that were susceptible to VVC (i.e., CBA, C57BL/6, A/J, C3H, BALB/c), as well as CD-1 mice that exhibit relative resistance to chronic fungal colonization/infection due to a diminished responsiveness to exogenous estrogen (36–38). We first sought to identify the signals responsible for initiating the inflammatory response. These studies showed that the transition of *C. albicans* from yeast to hyphae was required to trigger vaginal epithelial cells to produce the pro-inflammatory cytokines (IL-1β) and alarmins such as S100A8 that promote PMN migration into the vagina (39, 40). Brian Peters, our former postdoc and now a leading investigator in fungal pathogenesis, including VVC, conducted transcriptomic studies that further identified a role for the NLRP3 inflammasome and IL-1β signaling in the hallmark inflammatory response during VVC (40). It was subsequently revealed that candidalysin, the newly identified invasive toxin produced by *C. albicans* hyphae (41), was even more critical than hyphal formation itself for the induction of the immunopathogenic response (42, 43). These findings were built on seminal studies by the laboratories of Julian Naglik and Bernard Hube that identified candidalysin and defined key epithelial signaling events underlying tissue damage during mucosal *Candida* infections (44–46). Concurrently, we evaluated the putative roles for several pattern-recognition receptors (PRRs) and associated inflammatory mediators in the host response. After extensive studies employing PRR-deficient and S100A8-deficient mice, we ultimately found substantial redundancy among these pathways, with no single receptor or inflammatory mediator as indispensable for the inflammatory response (47). Another area of considerable investigation in the field at the time was the role of the Th17 axis in the host response to VVC. Considerable controversy surrounded the role of Th17 immunity, with our laboratory showing, through studies using knockout mice, that components of the IL-17 signaling pathway were dispensable for vaginal immunopathology, while studies by several other groups suggested a role for the Th17 axis (48–50). These discrepant findings were ultimately reconciled through collaborative work by Sarah Gaffen, Brian Peters, and colleagues demonstrating functional synergy among several IL-17-related cytokines in controlling vaginal fungal burden (51). Collectively, the complex interplay of host immune mechanisms and fungal virulence factors that drive VVC immunopathogenesis, at least in part, explained why efforts to identify roles for individual immune components of the response via inhibition/deletion led to negative results.

Additional studies further refined our understanding of the inflammatory process. We observed evidence of vaginal mucosal biofilm formation during infection, suggesting that *C. albicans* was present within the vaginal compartment in a manner that could favor persistence, host immune evasion, and resistance to antifungals (52–54). We also demonstrated that PMN recruitment to the vagina occurred independent of leukotriene signaling (55), indicating that alternative signaling pathways were capable of driving the robust PMN migration characteristic of VVC. Yet despite these advances in understanding the triggers and regulation of vaginal inflammation, a central paradox remained unresolved. Why did the abundance of infiltrating PMNs fail to reduce fungal burden?

Our continued efforts to answer this question eventually redirected our attention to the vaginal environment itself. Earlier work had suggested that epithelial cells possessed a modest protective capacity as epithelial cell-derived annexin A1 was shown to inhibit the growth of *C. albicans in vitro* (27, 56). While this fungistatic activity may contribute to broad mucosal defense, it was clearly insufficient to account for protection during vaginitis, particularly during intense inflammatory conditions. Therefore, we hypothesized that factors present within the vaginal microenvironment were acting to negatively influence PMN function.

Results from a number of subsequent exploratory studies finally revealed that the PMN dysfunction was attributed to the vaginal presence of heparan sulfate (HS), which served as a competitive inhibitor of Mac-1 on PMNs that is required for the interaction with Pra1 on *Candida* for the killing response (57). Hence, we proposed that the PMNs were functionally normal, but could not function properly in the vaginal environment due to HS. We termed this “neutrophil anergy” and ultimately confirmed this by several mechanistic approaches (58). First, we showed that the normal *in vitro* killing of *Candida* by PMNs in normal standard conditioned medium was ablated when assayed in vaginal conditioned medium (VCM) consisting of native vaginal secretions collected from lavages. Second, PMNs could kill effectively if heparanase was present to degrade HS in VCM. Third, we showed reduced killing by PMNs in normal conditioned medium spiked with purified HS. Interestingly, the resistant CD-1 mice had no evidence of HS in the vaginal environment, and hence, we proposed that the observed clearance of infection in CD-1 mice was at least in part due to infiltrating PMNs that retained their ability to effectively kill *Candida*. This was indeed confirmed by a subsequent study that revealed a clear NETosis-associated killing response in the resistant CD-1 mice C and no evidence of NETosis in VVC-susceptible mice (54). More recently, studies by Duncan Wilson and colleagues extended these concepts by showing that exogenous zinc can downregulate fungal Pra1 expression, reducing neutrophil recruitment and vaginal inflammation during VVC (59, 60).

Therefore, after almost three decades of intense research, we, together with contributions from several other laboratories, finally solved the long-standing riddle underlying the pathogenesis of VVC/RVVC as an immunopathogenic response with PMN dysfunction within the vaginal environment resulting in symptomatic VVC. The seminal clinical study opened the doors to solving the riddle as an immunopathogenic response, and the ensuing studies in the animal model forged the path to ultimately understanding the mechanisms associated with the immunopathogenesis that had been an enigma for so long. The key advances from the Fidel lab that collectively led to the current immunopathogenic model of VVC are summarized chronologically in Table 1. Together, these discoveries converged on the immunopathogenic model shown in Fig. 1, which provides a mechanistic framework for understanding host susceptibility and resistance to VVC, primarily from the animal model. In the end, we challenged dogma and contributed to a paradigm shift that now serves as a catalyst for immunotherapeutic approaches to reduce susceptibility to VVC.

**Fig 1:**
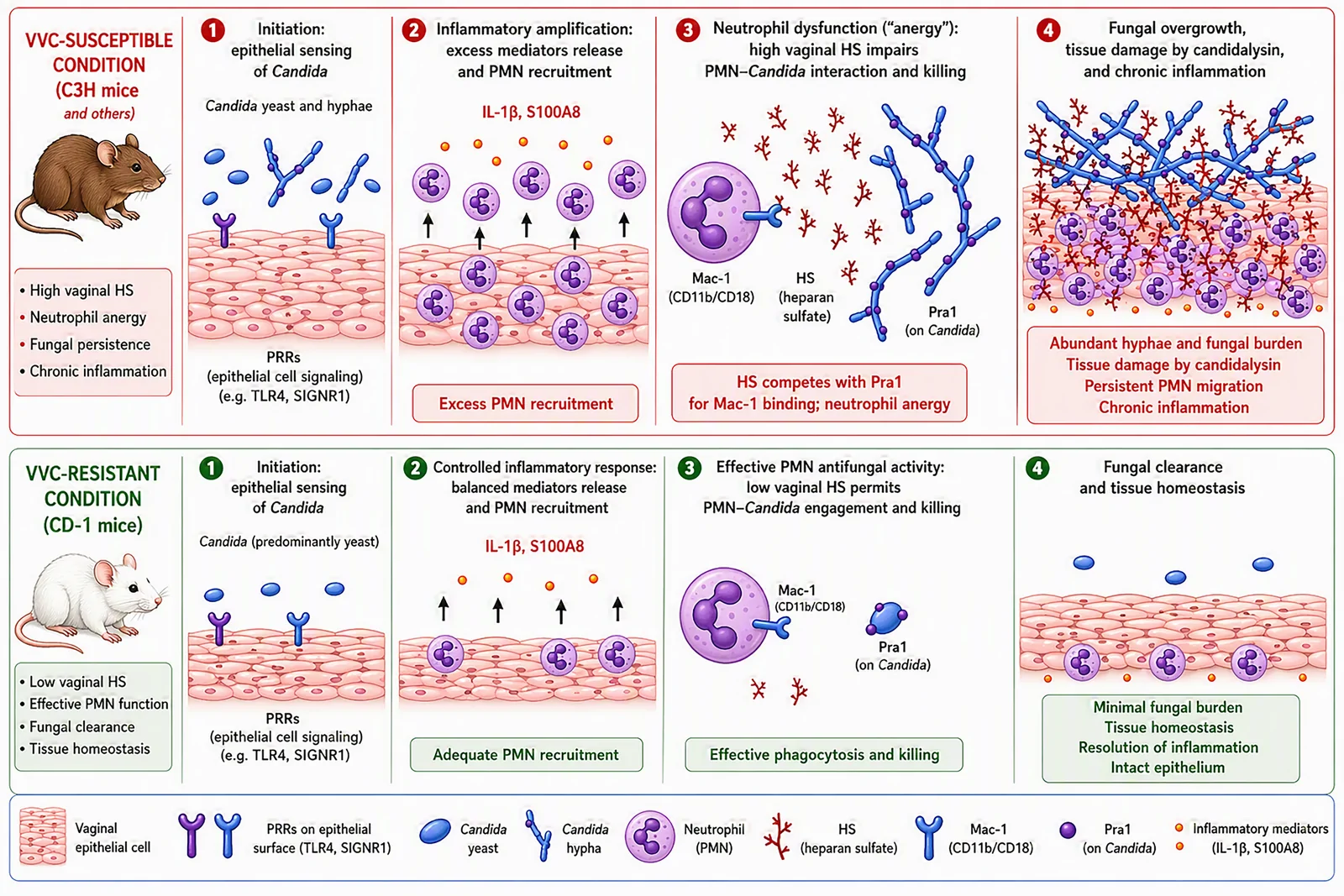
Proposed immunopathogenic progression of susceptibility and resistance to VVC. In VVC-susceptible hosts (e.g., C3H mice, upper panels), vaginal epithelial recognition of *Candida* occurs through pattern-recognition receptors (PRRs), including TLR4 and SIGNR1 (step 1). Epithelial activation promotes the release of inflammatory mediators, such as IL-1β and S100A8, leading to robust recruitment of polymorphonuclear neutrophils (PMNs) into the vaginal lumen (step 2). Despite substantial PMN migration, elevated vaginal heparan sulfate (HS) serves as a competitive inhibitor that interferes with the interaction between Mac-1 on PMNs and Pra1 expressed on the *Candida* surface, resulting in impaired antifungal activity (neutrophil anergy) (step 3). Consequently, fungal growth persists, hyphal burden increases, candidalysin-mediated tissue damage continues, and chronic inflammation is sustained with continued PMN migration (step 4). In contrast, VVC-resistant hosts (CD-1 mice, lower row panels) exhibit lower vaginal HS levels. Hence, following epithelial recognition of *Candida* and similar production of inflammatory mediators and PMN migration (steps 1 and 2), the low vaginal HS allows for effective Mac-1–Pra1 binding, preserving normal PMN-mediated phagocytosis and killing of *Candida* (step 3). Effective fungal clearance leads to resolution of inflammation, maintenance of epithelial integrity, and restoration of tissue homeostasis (step 4).

**TABLE 1:** Chronological milestones leading to the current immunopathogenic model of vulvovaginal candidiasis (1990–2026)

Period	Key advances	Major findings	Ref.
1990–2003	Lack of adaptive immunity in protection	Animal models and clinical studies demonstrated no significant role for T cells or antibodies in protection against VVC. Immunoregulatory mechanisms are responsible for the lack of adaptive immunity in the vagina. No adaptive immune deficiencies were identified in women with RVVC.	(5, 20, 21, 23–25, 48, 61–68)
1999–2005	Innate resistance by vaginal epithelial cells	Vaginal epithelial cells exhibited direct anti-*Candida* activity, while epithelial protection alone was insufficient to explain host resistance during active vaginitis. Epithelial cell-derived annexin A1 contributed to fungistatic activity and represented a frontline innate defense mechanism.	(26–31, 56)
2002–2004	Clinical identification of an immunopathogenic response	A live intravaginal *Candida* challenge study in women demonstrated that symptomatic infection was associated with a massive neutrophil (PMN) infiltrate, whereas women who remained asymptomatic showed little or no cellular infiltration. This study led to the paradigm-shifting hypothesis that VVC pathogenesis reflects an immunopathogenic inflammatory response rather than an immune deficiency.	(35)
2004–2021	Mechanisms initiating immunopathology	*C. albicans* transition from yeast to hyphae and candidalysin was necessary to trigger epithelial cell activation. Vaginal epithelial cells produced IL-1β and S100A8, promoting PMN recruitment into the vagina. Select PRRs, including TLR4 and SIGNR1, participated in vaginal epithelial sensing with considerable redundancy among multiple pro-inflammatory factors involved in the immunopathogenic processes.	(39, 40, 43, 47, 69)
2010–2024	Additional factors contributing to persistent inflammation	Evidence supported vaginal mucosal biofilm formation by *C. albicans*. PMN recruitment occurred independently of leukotriene signaling pathways. In resistant CD-1 mice, NETosis-mediated antifungal activity contributed to fungal clearance, whereas susceptible strains lacked effective NETosis responses.	(53–55)
2014–2017	Mechanism of neutrophil dysfunction (neutrophil anergy)	PMNs remained intrinsically functional but were inhibited within the vaginal environment of susceptible hosts. Vaginal heparan sulfate (HS) was identified as a competitive inhibitor of the Mac-1/Pra1 interaction required for PMN-mediated killing of *Candida*. Heparanase restored PMN killing by degrading HS in mouse and human vaginal conditioned media (VCM).	(57, 58)
2023–2026	Clinical validation/translational studies	Women with RVVC exhibited elevated vaginal HS, which impaired PMN killing, providing mechanistic validation of the immunopathogenic model in humans. HS degradation by heparanase (HPSE) in vaginal secretions enabled restoration of PMN antifungal activity. Intravaginal cooling reduced immunopathology and symptom burden.	(58, 70)

Although this article focuses on the story behind the science of the Fidel lab, many other investigators made important contributions that also shaped or extended the understanding of VVC pathogenesis. These advances include studies of host responses to *C. albicans*, *C. albicans* virulence factors, roles for non-*albicans Candida* species, *C. albicans*–epithelial cell interactions, host genetic susceptibility to RVVC, and novel therapeutic approaches. We particularly acknowledge the contributions of Sarah Gaffen, Brian Peters, Duncan Wilson, Mihai Netea, Julian Naglik, Bernhard Hube, Robert Wheeler, Jacques Ravel, Anna Vecchiarelli, Eva Pericolini, Luigina Romani, Christophe d’Enfert, Jack Edwards, Antonio Cassone, Flavia De Bernardis, and many others whose work refined the concepts described in this article and continue to shape the field today.

Solving the riddle was obviously a rewarding highlight and the heart of the “story behind the science” for our laboratory. But there was one last study that solidified the immunopathogenesis of VVC and brought the entire field full circle. Accordingly, we reasoned that it was critically important to evaluate whether the same immunopathogenic response identified in the mouse model could also be demonstrated clinically during RVVC. Specifically, we sought to determine whether the PMN infiltration shown in that seminal study without evidence of fungal clearance was similarly associated with the presence of vaginal HS. To address this question, another seminal study was initiated in collaboration with our long-time colleague, Jack Sobel at Wayne State University. Indeed, the clinical study showed strong evidence for HS within the human vaginal environment during episodes of RVVC. Similar confirmation studies to those performed in the mouse model were conducted using human VCM collected from women with active RVVC, women in remission from RVVC while on fluconazole maintenance therapy, or normal control women with no history of VVC. Experiments included quantification of HS in VCM samples, testing human PMN antifungal activity in the various VCM preparations, use of heparanase to degrade putative HS in inhibitory (RVVC) VCM, and spiking non-inhibitory VCM (RVVC remission/controls) with exogenous HS to show reduced PMN killing activity (58). The results all pointed to the similar presence of HS in the vaginal environment of women with RVVC that promotes a PMN dysfunction that allows for persistance of *Candida* while contributing to the symptoms associated with VVC. Thus, we concluded a full-circle mechanistic confirmation of the immunopathogenesis of VVC, both clinically and in the mouse model.

Ultimately, these studies should indeed serve as a catalyst for immunotherapeutic approaches to reduce the symptoms associated with VVC/RVVC and/or promote PMN-mediated fungal clearance. Importantly, our findings highlight a viable therapeutic target, HS, and the potential use of heparanase (HPSE) to degrade HS, reverse PMN dysfunction, and restore fungal killing. Combined with strategies that reduce fungal burden (antifungal drugs) or revert *Candida* hyphae to blastoconidia to reduce the triggers for the immunopathogenic response, these approaches should reduce or eliminate the symptoms associated with the infection. The latter concept has recently been shown using cold therapy (70). In parallel, conventional antifungals continue to be optimized, while repurposed agents, alternative formulations, and combination approaches are being explored to improve efficacy and reduce recurrence (71–77). Of course, a vaccine that keeps vaginal *C. albicans* in the commensal state would be ideal. Several vaccine strategies have been explored, including the *C. albicans* adhesin agglutinin-like sequence 3 (Als3)-based vaccine (NDV-3A, Novadigm, USA), developed by Jack Edwards, Ashraf Ibrahim, and colleagues (78), and the recombinant secretory aspartyl proteinase 2 (rtSap2)-based vaccine (PEV7, Pevion Biotech, Switzerland), pioneered by Antonio Cassone and colleagues (79), with the NDV-3A vaccine showing modest yet significant efficacy in reducing symptoms in women with RVVC. More recent preclinical approaches include novel candidates such as the “pan-fungal” NXT-2 vaccine (80) and candidalysin-targeted nanobodies (81), developed by the groups of Karen Norris and Bernhard Hube, respectively. A newer investigational vaccine, Candi5V (LimmaTech Biologics, Switzerland), is a uniquely engineered pentavalent bioconjugate designed to elicit both humoral (Th2) and cellular (Th1/Th17) responses against RVVC (77). In the end, antifungal therapies in conjunction with immunotherapies or vaccines should ultimately maintain *C. albicans* in the harmless commensal state that is integral to vaginal homeostasis.
